# Education Research: Evaluating Medical Student Learning Preference and Its Relationship to Clerkship Satisfaction

**DOI:** 10.1212/NE9.0000000000200102

**Published:** 2023-12-06

**Authors:** Margo A. Peyton, Andrew S. Lea, Roy E. Strowd, Rachel Marie E. Salas, Charlene E. Gamaldo, Doris G. Leung

**Affiliations:** From the Department of Neurology (M.A.P.), Mass General Brigham; Department of Medicine (A.S.L.), Brigham and Women's Hospital, Boston, MA; Department of Neurology (R.E.S.), Wake Forest School of Medicine, Winston-Salem, NC; and Department of Neurology (R.M.E.S., C.E.G., D.G.L.), Johns Hopkins University School of Medicine, Baltimore, MD.

## Abstract

**Background and Objectives:**

The Accreditation Council for Graduate Medical Education and the Association of American Medical Colleges' commitment to competency-based medical education (CBME) has shifted the medical education landscape. Education methods conducive to CBME are learner-centered and give educators the opportunity to develop a more personalized approach to curricular development and delivery. By understanding learning preferences, educators are better positioned to respond to the changing needs of students. The Learning Preference Inventory (LPI) is a validated tool that assesses preferences across 3 domains: (1) content delivery (concrete vs abstract), (2) instruction (teacher-centered vs student-centered), and (3) learning structure (individual vs interpersonal). Using the LPI, our objective was to describe the learning preferences of medical students in the Neurology clerkship and to evaluate how preferences correlate with satisfaction with curricular elements.

**Methods:**

A cohort of second-year through fourth-year medical students rotating through the required Neurology clerkship at a single institution was identified. All students completed the LPI and a survey to assess satisfaction with curricular elements, including an in-person localization session, virtual simulation, and a summative case study.

**Results:**

Five hundred thirty medical students were included. Preference for concrete content delivery was much more common than abstract (83% vs 17%); otherwise students were evenly divided in their preferences for teacher-centered vs learner-centered instruction and interpersonal vs individual learning structure. There was a significant difference in LPI responses across medical school years: More third-year and fourth-year students preferred individual learning structure compared with second-year students (*p* = 0.040). Learning preferences also correlated with course satisfaction survey responses. Student satisfaction with the course activities was overall positive, with 69% of students agreeing that the course enhanced their learning. Abstract learners were significantly more likely to report that the virtual simulation enhanced their learning than concrete learners (*p* = 0.018).

**Discussion:**

Neurology clerkship students demonstrated clear learning preferences that were associated with satisfaction with specific curricular content. There were significant differences in learning preferences across medical school years, suggesting that learning preferences may shift throughout training and may be influenced by clinical exposure. In an educational environment that cultivates the success of all learners, the LPI provides important data to inform curricular development and achieve personalized medical education.

## Introduction

The Accreditation Council for Graduate Medical Education and the Association of American Medical Colleges' (AAMC) commitment to competency-based medical education (CBME) has driven a shift in the medical education landscape.^1^ CBME uses an outcomes-focused assessment approach to ensure residency preparedness and improve patient care. Education methods conducive to CBME are learner-centered and active, such as case-based and problem-based learning, because they engage students with complex problems that mirror the challenges of residency and cultivate lifelong learning.^2^

Compared with lecture-based instruction, learner-centered methods allow educators to develop a more personalized approach to curricular development and delivery. Different educational activities such as group projects, student-led presentations, and role play exercises engage students to different degrees depending on the students' preferred learning method. In knowing how their students learn best, educators can incorporate learner-centered methods that engage their students' preferences. Medical educators have used surveys like the VARK questionnaire, Kolb's Learning Style Inventory, and Felder and Soloman's Index of Learning Styles to evaluate learning style and compare the results with examination performance or satisfaction with the curriculum.^3,4^ However, in medical education, learning style has not been shown to correlate with examination performance or other learning outcomes.^3-5^ Educational theory reinforces this finding, arguing that “styles are not specific to particular tasks and no one value can be assigned to a style.”^6^ Essentially, for any task, there is no definitionally more successful style than another.

The Learning Preference Inventory (LPI), developed in 1974 with health professional students and practitioners, is a validated tool to assess learning preferences.^7^ The LPI helps educators understand medical learners and inform teaching methods unique to the medical education and work environment. The LPI differs from the aforementioned inventories in 2 critical ways. First, the LPI is an assessment of learning *preferences* rather than learning *styles*—an important distinction given recent literature debunking previous notions about the value of learning styles.^8^ Whereas the learning styles hypothesis refers to the refuted idea that learning improves when the type of instruction meshes with the student's learning style, learning preference simply refers to the observation that learners reliably volunteer preferences on how they receive instruction. Second, the LPI is specific to the educational environment of medicine and health care. Rezler and Rezmovic define learning style as how “an individual perceives and processes information in learning situations” and learning preference as “the choice of one learning situation or condition over another.”^7^ This aligns with the work of Lynn Curry, PhD, whose learning theory sought to define learning style as independent of learning environment and learning preference as dependent on learning environment.^9^

Previous work involving the LPI has compared medical student learning preferences with curricular satisfaction and with performance on National Board of Medical Examiners (NBME) subject examinations. A study of 94 preclinical medical students who took the LPI found that more students were abstract compared with concrete and individual compared with interpersonal learners, and that abstract and individual learners most benefitted from problem-based learning.^10^ A study of 116 clinical medical students who took the LPI and a survey to assess their satisfaction with live compared with virtual instruction found that more students were concrete learners, and that abstract compared with concrete learners were more satisfied with virtual than live instruction and vice versa. Like the learning style studies mentioned above, the study also found that learning preferences did not correlate with examination performance.^11^

The theoretical difference between the environment independence of learning style and environment dependence of learning preference yields an opportunity to examine these qualities in medical training. In comparing LPI responses across medical years, we can ask a new question: Does exposure to the clinical environment change learning preferences? Our study also seeks to expand the use of the LPI, a less commonly selected measure for learning style/preference of medical students, but one best suited for health professionals and medical environments. We aimed to demonstrate that the LPI can be used to inform the development of curricula that foster student satisfaction and engagement.

## Methods

### Study Population

We sought to understand the learning preferences of medical students enrolled in the neurology clerkship at a single institution and determine whether these preferences correlated with learner satisfaction with the curriculum. The neurology clerkship is a required, 3-week clinical experience in which second-year through fourth-year medical students rotate with teams of residents and attendings. Medical students do not select when they complete the clerkship (i.e., second, third, or fourth year) and are randomly assigned by medical school administration. We administered the LPI to second-year, third-year, and fourth-year medical students from 2015 to 2020 (before curricular changes related to coronavirus disease 2019 [COVID-19]) and a survey to assess satisfaction with 3 curricular activities.

### Survey Methods and Outcome Variables

**Figure 1:**
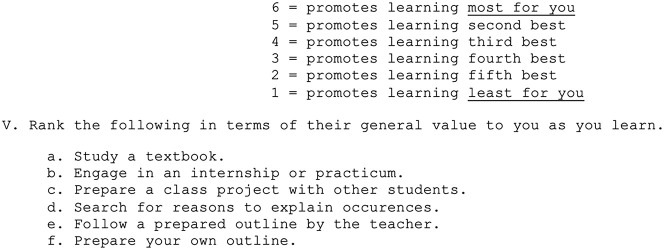
LPI Survey Sample Question A sample prompt from the LPI in which learners rank options a-f, 6 for most valued and 1 for least valued.

The LPI assesses preferences across 3 domains: (1) content delivery (concrete vs abstract), (2) instruction type (teacher-centered vs student-centered), and (3) learning structure (individual vs interpersonal) (Table).^7^ Examples of each are as follows and are further elucidated in the Table: (1) perform a procedure (concrete) vs study theory (abstract), (2) follow an outline from the teacher (teacher-centered) vs students write their own outlines (student-centered), and (3) study alone (individual) vs study with others (interpersonal). To determine their specific preferences, learners rank 6 statements from “promotes learning least for you” to “promotes learning more for you” in response to a series of prompts. For instance, a prompt asks: “Rank the following in terms of their general value to you as you learn.” Some of the statements to rank include “Study a textbook,” “Engage in an internship,” or “Prepare a class project with other students” (Figure 1). Learners then add up their ranked responses in a specific manner to determine whether they more often prefer concrete or abstract information, teacher-centered or student-centered instruction, and individual or interpersonal settings.

**Table:** Learning Domains of the LPI

Learning preference domains	Preference for:
Content delivery	
Concrete	Tangible, specific, practical tasks, with focus on skills
Abstract	Theories and generating hypotheses with focus on general principles and concepts
Instruction	
Teacher-centered	Well-organized teacher-directed class, with expectations, assignments, and goals clearly identified
Student-centered	Student-organized tasks, with emphasis on autonomy and self-direction
Learning structure	
Individual	Learning or working alone, with emphasis on self-reliance and solitary tasks such as reading
Interpersonal	Learning or working with others, with emphasis on harmonious relations between students and teacher and among students

Abbreviation: LPI = Learning Preference Inventory.

The 3 learning domains of the LPI with descriptions of each preference as defined in the original 1981 article.

To examine the correlation between learning preferences and satisfaction with curricular activities, we needed to use a clerkship curriculum with varied instructional design and delivery methods. Clinical Reasoning in Neurology (CRN) is a curriculum developed for students rotating through a neurology clerkship at the Johns Hopkins School of Medicine.^12^ While bedside clinical teaching provides the foundation of medical education for clerkship students, formal instruction provides necessary and complementary standardized learning.^13^ While the clinical learning environment can present variable pathology, CRN ensures that students learn foundational topics in neurology. CRN provides instruction of neuroanatomy, diagnosis, and management through an online, interactive, and flipped classroom style workshop series of 3 case-based learning activities.

**Figure 2:**
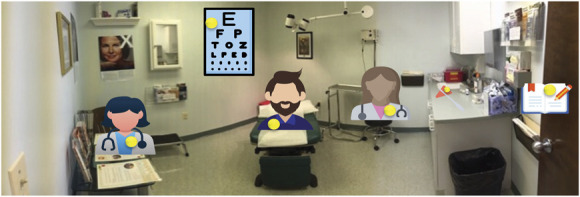
Virtual Patient Simulation Interface The virtual examination room of the virtual patient simulation in which learners collect signs and symptoms and then complete a series of questions.

**Figure 3:**
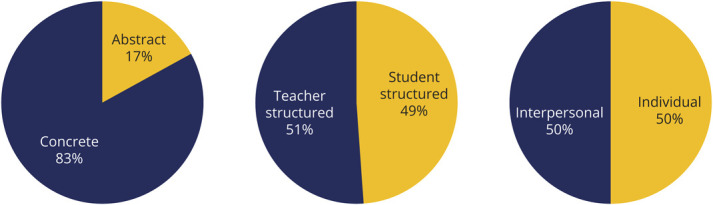
Learning Preferences of Neurology Clerkship Students Eighty-three percent of students preferred concrete to abstract learning, while teacher vs student and interpersonal vs individual preferences were more evenly divided.

The surveyed CRN activities included (1) a localization session, initially bedside then converted to a classroom setting in 2019, in which a neurology faculty member led an in-person group of students through a neurologic examination; (2) virtual patient simulation, in which students gathered clinical data in a virtual space and then completed short answer and multiple-choice questions (Figure 2); (3) summative case study, in which students reviewed a history and physical and then completed an assessment and plan virtually. The virtual patient simulation and summative case study assignments were “open book,” and students were allowed to use reference materials and to work in groups if they wished. In these 3 activities, students were exposed to different methods of content delivery, instructional type, and learning structure, facilitating the analysis of learner preference and satisfaction across medical school clinical years. On submission of their summative case study assignments, students were directed to an online survey with Likert-type responses to rate their satisfaction with individual CRN activities and the CRN curriculum as a whole.

### Statistical Analysis

An exploratory analysis of collected variables was performed using frequency tables and histograms. For learning preference domain scores, Shapiro–Wilk tests and quantile–quantile plots were used to assess whether variable distributions met the assumption of normality. Learning preference domain scores were also stratified into dichotomous variables (i.e., more concrete vs more abstract) to allow analysis as categorical variables. Pearson χ^2^ tests were used to compare categorical variables and ordinal variables (including medical school year and survey response items). The statistical significance threshold was set at <0.05.

### Standard Protocol Approvals and Registrations

The study qualified for an exemption per the Johns Hopkins Institutional Review Board. The analyzed data set did not contain data on student demographics, except for year in medical school, or performance on clinical evaluations or NBME subject examination.

### Data Availability

Anonymized data will be shared by request from any qualified investigator.

## Results

From a cohort of 569 second-year, third-year, and fourth-year medical students, 530 completed the LPI at the beginning of the clerkship, representing a 93% response rate. Fifty-eight percent (309) of students who completed the LPI were third-year medical students (MS3s), followed by 23% (121) fourth-year students (MS4s), and 19% second-year students (MS2s) (99). The most common learning preferences were overwhelmingly concrete (83%, 440) (vs abstract), followed by near even division of teacher-centered (51%, 270) (vs student-centered), and interpersonal (50%, 262) (vs individual) (Figure 3). There were no significant differences in learning preferences over the 5-year period of the study, and data from after the curricular changes related to COVID-19 were not included.

**Figure 4:**
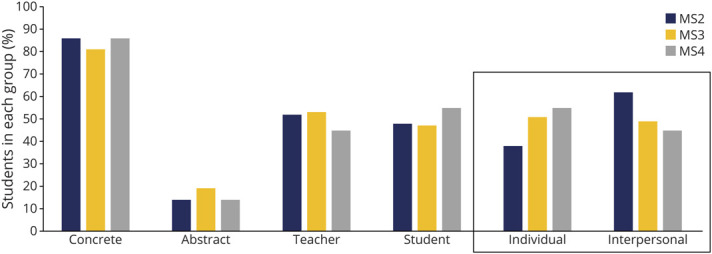
Learning Preference Differences Across Years in Medical School Significantly more third-year and fourth-year students were individual learners compared to second-year students (*p* = 0.040). The percentages represent an exact count of students in each group.

There was a significant difference in learning preferences across medical school years. For learning structure, MS3s and MS4s, compared with MS2s, had higher individual scores than interpersonal scores: 51% (158) of MS3s and 54% (66) of MS4s, compared with 38% (38) of MS2s (*p* = 0.040) (Figure 4). This correlated with a corresponding downtrend in interpersonal learning preferences with each advancing year in medical school training. For content delivery and instruction type, there were no significant differences for abstract compared with concrete and teacher-centered compared with student-centered learning, across medical school years.

The overall response rate for the CRN satisfaction survey was 89% (506). Response rates were similar across medical school years (86% for MS2s, 91% for MS3s, and 85% for MS4s). The response rate for each academic year ranged from 74% to 99%. The lowest response rate occurred in the 2019–2020 academic year, most likely because the suspension of in-person learning due to the COVID-19 pandemic occurred before students on the clerkship completed the CRN activities. Student satisfaction with the CRN activities was overall positive, with 69% (349) of students responding “agree” or “strongly agree” when asked whether the CRN course strongly enhanced their learning. Sixty-four percent (324) of respondents agreed or strongly agreed that the CRN helped them to approach the evaluation of patients on the clerkship. When asked if they would recommend the CRN to a colleague, 59% (299) responded that they “agree” or “strongly agree.”

There was no difference in student satisfaction with most of the curricular elements based on learning preference. However, students with greater abstract scores rated the virtual simulation more highly than students with greater concrete scores. When asked about the virtual simulation, 62% (48) of more abstract learners reported that it enhanced their learning compared with 51% (201) of more concrete learners (*p* = 0.018). For comparison, 53% (268) of all respondents agreed or strongly agreed in answer to this question. When stratified by medical school year, abstract learners rated the course more highly than concrete learners in each year, although the association remained statistically significant for the MS2 year only. There were no significant differences in curricular satisfaction survey responses for teacher-centered compared with student-centered learners or for individual compared with interpersonal learners.

## Discussion

Medical students in the neurology clerkship demonstrated clear learning preferences associated with satisfaction with specific CRN activities. The dominance of concrete learning preference for content delivery may reflect the skills typically associated with premedical academic success. Prerequisite coursework for medical school emphasizes the concrete application of abstract concepts in the form of problem sets and laboratory work. Extracurricular activities associated with premedical studies, such as shadowing, volunteering, and research, also consist of concrete tasks. This explanation, however, ignores the growing number of students who pursue fields of study less classically associated with medical prerequisites or have work experience outside of medicine. In addition, it is important to consider that the CRN is an adjunct to the clinical learning environment, an inherently concrete delivery method.

The significant difference in learning preference across medical school years suggests that learning preferences may not remain constant throughout training and may be influenced by clinical exposure. The theory behind this result of our study hinges on the earlier discussion of style vs preference and the degree of influence of the learning environment. In their study involving the LPI, Chapman and Calhoun sought to validate a learning style hypothesis originally proposed by Curry to clarify the abundance of learning style terminology and methodologies.^9,10^ Curry argued that there were 3 levels of learning styles, from most static to most mutable: cognitive personality style, information processing style, and instructional format preference. While she cited the LPI as an example of an assessment of instructional format preference (most influenced by the learning environment), she noted the content delivery domain of the LPI to be less dependent on “social conditions.”^9,14^ Consequently, whether a learner is concrete or abstract may be closer on the continuum to style (static) than to preference (mutable).

Despite the shift in environment from preclinical to clerkship learning, we only found one of the LPI learning domains (learning structure) to be unstable across medical school years. It is possible that the learners in our study found that the learning structure domain, compared with content delivery and instruction type domains, changed the most from preclinical to clerkship learning. MS3s and MS4s, with comparatively more clinical experience than MS2s, may prefer individual learning because of the time and place constraints of clerkships, when they may not have the same schedules and placements as their peers to facilitate group work. For educators, the increasing independence of MS3 and MS4 learners suggests there may be value in allowing more self-directed clerkship learning.

Finally, the finding that learners with higher abstract scores were more likely to agree that the virtual patient simulation enhanced their learning may seem unexpected because simulation is an exemplary concrete activity. Compared with the summative case study, however, the virtual simulation required that students gather clinical data themselves, rather than receiving a completed history and physical for review. The activity also required that students complete short answer questions, in addition to multiple-choice, that do not necessarily have a single correct answer. While a concrete exercise, the virtual simulation may have had comparatively more intellectual flexibility to attract more abstract learners.

More broadly, the correlation between learning preference and curricular satisfaction advocates for increased attention to the learner experience, which is assessed yearly with AAMC's Graduation Questionnaire. When learners prefer an activity's delivery, instruction, or structure, it has the potential to increase engagement and motivation.

Limitations of our study include a small sample size (n = 569) at a single institution. In addition, the data contain a self-reported inventory and no academic performance data. We also did not collect data on whether students completed the assignments alone or in groups, which may have influenced satisfaction for students with different learning preferences. While statistically significant difference in leaner satisfaction was identified with respect to learner preference, there are limited data to determine how meaningful these differences are in this population.

Next steps in our research are 2-fold. Our data reflect student learning preferences and curricular satisfaction before the curricular changes that resulted from COVID-19. As in-person clinical experiences and CRN curricular activities have resumed, there may be value in continuing to assess learning preference and curricular satisfaction to gauge the impact of remote learning during COVID-19. There has also been interest in whether learning preferences align with residency specialty choice. While Curry's designation of the LPI as an assessment of mutable characteristics suggests it may not be the best tool to assess what is more likely a cognitive personality question, administering the LPI to residents may help guide graduate medical education curricular development.

In a learner-centered educational environment that cultivates the success of all medical students, the LPI provides educators with important data to inform curricular development and achieve personalized medical education. Our results showed that students demonstrated clear learning preferences associated with satisfaction with curricular activities. The CRN activities had qualities of content, instruction, and structure to study all possible preferences. While it is unreasonable to customize a curriculum for each individual learner, offering a curriculum with varied design and delivery methods may engage more learners and prepare them for the next stage of medical training.

## Appendix Authors

**Table:**

Name	Location	Contribution
Margo A. Peyton, MD	Department of Neurology, Mass General Brigham, Boston, MA	Drafting/revision of the manuscript for content, including medical writing for content; study concept or design; analysis or interpretation of data
Andrew S. Lea, MD, D.Phil	Department of Medicine, Brigham and Women's Hospital, Boston, MA	Drafting/revision of the manuscript for content, including medical writing for content; study concept or design; analysis or interpretation of data
Roy E. Strowd III, MD, MEd, MS	Department of Neurology, Wake Forest School of Medicine, Winston-Salem, NC	Drafting/revision of the manuscript for content, including medical writing for content; major role in the acquisition of data; study concept or design; analysis or interpretation of data
Rachel Marie E. Salas, MD, MEd	Department of Neurology, Johns Hopkins University School of Medicine, Baltimore, MD	Drafting/revision of the manuscript for content, including medical writing for content; major role in the acquisition of data; study concept or design; analysis or interpretation of data
Charlene E. Gamaldo, MD	Department of Neurology, Johns Hopkins University School of Medicine, Baltimore, MD	Drafting/revision of the manuscript for content, including medical writing for content; major role in the acquisition of data; study concept or design; analysis or interpretation of data
Doris G. Leung, MD, PhD	Department of Neurology, Johns Hopkins University School of Medicine, Baltimore, MD	Drafting/revision of the manuscript for content, including medical writing for content; major role in the acquisition of data; study concept or design; analysis or interpretation of data

## Glossary

AAMC: Association of American Medical Colleges
CBME: competency-based medical education
COVID-19: coronavirus disease 2019
CRN: Clinical Reasoning in Neurology
LPI: Learning Preference Inventory
NBME: National Board of Medical Examiners
